# On the Use of Chlorate of Potash and Glycerine Injections for Ulcerations in Chronic Dysentery

**Published:** 1873-10

**Authors:** 


### Of the use of Chlorate of Potash and Glycerine Injections for the Ulcerations in Chronic Dysentery.

Under the above tltle Dr. Mead gives (New York Medical Journal, Sept., 73,) a report of two cases of Chronic Dysentery treated by this method. We copy his remarks in full and the report of Case I.

Probably one of the most intractable of the various diseases that inflict humanity is chronic dysentery. It will, undoubtedly, take a prominent place in the medical history of the late civil war, as far as camp diseases are concerned. The disease is essentially a chronic ulceration of the mucous membrane of the rectum and colon, and may continue the entire length of both, but is usually confined to the former. Ulcerations may, however, exist in the smaller intestines, but with these I have nothing to do at present, nor with any of the ulcerations produced by scrofulosis, cancer, or as sequelæ of syphilis.

The disease in the first instance is undoubtedly produced by some irritant coming in contact with the part, after the general health has been undermined by the acute dysentery, or it may be by typhoid fever; and I deem it not impossible for the ulcerations to be superinduced by any disease that may depress the general health, together with the application of the irritant.

This irritant may be any thing that will induce an inflammation of the mucous membrane. Probably the acrid secretions of the intestines themselves produce more cases than all other causes. Violent and long-continued purging with drastic cathartics may prove the cause; corrosive sublimate is also mentioned as a possible cause. It may, perhaps, be a question whether the ulcerations of Peyer's patches in enteric fever can result in chronic ulceration of the large intestine. Whatever may be the cause, the subsequent history is the same. The ulceration that takes place in the first instance is substantially the same that we sometimes see in the mouth and fauces, produced by some disorder of digestion or other cause, and would be equally amenable to treatment could we but make the same direct application of our remedies, and prevent the continued presence of the original or some other irritant. The subsequent history of the ulcerations in the two locations, provided there be no medication, is very different. Those of the mouth will probably recover, while those of the colon and rectum will probably continue to grow worse.

This tendency is probably the result of the bad condition of the general health, the presence of unhealthy acrid fæces, the nervous irritation of the inflammation, the frequent desire to go to stool. and tenesmus; the straining and contractions of the muscular coats of the intestine, causing the rubbing together of the diseased and other portions of the bowel, thus increasing and spreading the ulcerations by the application of the diseased secretions to the otherwise healthy tissue. Sometimes the ulcerations, after a variable length of activity, seem to pass into a state of quiescence, and their surfaces become covered with a whitish membrane. During this state of quiescence, the patient often deceives himself into thinking he is getting the better of the disease; but, in a short time, owing, as he supposes, to some indiscretion on his part, either in exposing himself or indulging his appetite, he finds himself worse off than before. This attack, however, is produced by a renewed activity of the ulcers, without throwing off the membranes that have been formed over them, but by breaking either through them or by their sides. After another time of activity, they again become quiescent, and other covering membranes are formed over the first. The disease thus goes on, the membranes continue to thicken, until the patient dies from the continued drain on his system, or is cut off by some inter-current disease that under ordinary circumstances might be of but little moment. I have seen the coats of the colon and rectum from one-fourth to a third of an inch in thickness at the seat of ulceration, where the patient had died of this disease.

The treatment in this condition, usually recommended, consists in the use of opium, quinine, iron, alcohol, bismuth, astringents, a strict diet, etc. With what success these remedies have been used, let the tombstones and hospital death-records report. That medicine given by the mouth can have any decidedly remedial effect upon the ulcerations of the colon or rectum, is extremely questionable, and especially if the remedy be nitrate of silver, sulphate of copper, or any simple astringent.

Flint says ("Practical Medicine," third edition): "Chronic dysentery is one of the most intractable and hopeless of diseases. * * * The treatment of chronic dysentery relates first to the local affection. Remedies to allay irritation and to promote the healing of ulcerations are indicated. But, unhappily, in the great majority of cases, there is very little probability that a cure will be effected, and all that can be hoped for from judicious treatment are palliation of symptoms and prolongation of life."

The ulceration itself is purely a local disease, and should be treated locally, and, instead of astringents, a stimulant to the mucous membrane should be used. The objects of treatment should be to heal the ulcerations, and to induce such a healthy action of the part as to throw off the whitish membranes that have been formed over them. The remedies that have been found efficient in ulcerations of the mucous membrane of the mouth, etc., should, by a reasonable inference, be equally efficient for ulcerations in any other part of the alimentary tract. And such I have found to be the fact.

My treatment is, to inject into the bowel half a drachm of chlorate of potash rubbed up in half an ounce of glycerine, and mixed with three to four ounces of warm water, two or three times a day, the patient to be confined to the bed with instructions to hold the enema as long as possible. The first injection will not be held over half a minute, and not that length of time if the rectum be much affected. But in a few days the trouble in this respect will be greatly overcome, and, as the ulcerations heal, greater tolerance of the medicine will be evinced. After these injections have been used for from seven to ten days, the whitish membranes and *debris* of the ulcers, provided it be an old case, will be passed with the stools, looking like scraped lint. This occurred in the first case I had, to the no small surprise of my patient. The general health of the patient, of course, needed continued attention, and such medication as the case indicated was prescribed.

The two following cases I give as showing the result of this method of treatment:

CASE I.—Came under treatment June 4, 1868. D., aged twenty-seven years, light, fair complexion, light-colored hair, hazel eyes; formerly of Connecticut, and colonel of one of the regiments of volunteers during the late war. Was first attacked in July, 1861, while on duty with his regiment in Virginia. Afterward ordered to South Carolina, where he remained on duty until his health completely failed, and he was forced to resign and return home. From the time of his attack until I took charge of his case he had been under the care of good physicians, and had undoubtedly received the benefit of the best treatment as laid down by our authors. He informed me, however, that he had not had a natural stool since his first attack, and that all the effect the medicines seemed to have was simply to keep him alive. The amount of medicine that he had taken was astonishing, and especially so as he had lived through it all, saying nothing of the ravages of the disease. And, as may be supposed, his faith in the medical profession was as near *nihil* as possible, so far as his own case was concerned. At this time, June 4, 1868, he was having from twenty to thirty stools in the twenty-four hours, was in a very weak, anæmic condition, with hardly strength enough to stand alone. His muscles were atrophied and flabby, the skin dry, pulse very weak, and in all respects he appeared incapable of living more than a week. The general appearance of these patients just before death is too well known to need any further description of this one, as he was in all respects a typical and a classical one.

I commenced the use of injections at once, prohibited the use of opium and whiskey, which had always been ordered him in great quantities during the whole of his sickness, and were doing him much more injury than benefit. Subnitrate of bismuth, in forty-grain does, suspended in mucilage, three times a day, with quinine, iron, strong beef-tea, beefsteak, eggs, etc.; in fact, gave him the most powerful diet as to kind and quantity he could take, and leave the least amount of *debris* to pass the intestines. The injections at first were returned as soon as thrown up, but produced a decided impression upon him. It seemed to him, he said, "as though they would take his breath away," and the pain for some time was intense. But, in a short time, the unpleasant sensations became less prominent, and, although it was several days before he could hold the injection for an hour, yet the tenemus and desire to go to stool, together with the passage of mucus and pus, were all most sensibly improved within a very few days, and at the end of twelve days the stools were reduced to eight or ten in the twenty-four hours. He continued to improve in all respects until his health was restored, except a partial cirrhosis and functional disorder of the liver, produced by the long-continued use of alcoholic liquors that had been prescribed for him. There was a good deal of tenderness of the bowel, which continued for nearly two years, but, up to the present time, he has had no return of his old trouble that could not be controlled by hyoscyamus and tannate of bismuth, and has had no return of dysentery for at least two years. His stools are natural in all respects, his appetite good, and his diet as miscellaneous as one need wish. He was under treatment three months before he was able to resume his duties at the office and work all day.

The history of these two cases, before I commenced their treatment, was derived from the patients themselves, They are both intelligent men, have some knowledge of medicine, and are perfectly trustworthy in all their statements.

I am well aware that it is not safe to draw conclusions from a few successful cases treated in a different method from that advised by the standard authors; but, when they admit that their method nearly always proves fatal, we are certainly justified in endeavoring to discover some new plan, "and when found make a note of it."

———:o:———

We copy the report of the following remarkable case from the *Medical and Surgical Reporter*, Dr. S. W. Butler, Editor, for September, 1857. We have recently seen and conversed with Dr. Seaman concerning the case. He showed us the portions of bone removed, which correspond with the report below. Dr. Seaman had the kindness to present the specimens to the Buffalo Medical College Museum, where it will be treasured as a remarkable curiosity. He informs us that the patient is still alive and in full possession of all his faculties. A few months after the accident he married, and is now the father of five or six children. The eldest, a girl, for some time after her birth presented a nervous, frightened look, similar to that observed in the father by Dr. Seaman during his sickness. The patient complains of no pain or unnatural feeling in the parts, the only inconvenience experienced being a sense of fullness at the seat of injury when engaged in too active exertion, or when stooping over. Were it not for the fact that we are personally acquainted with Dr. Seaman, and have seen the fragments of bone removed, we should hesitate in presenting the case to our readers as being worthy of acceptance.—[ED.

